# RANKL^+^ senescent cells under mechanical stress: a therapeutic target for orthodontic root resorption using senolytics

**DOI:** 10.1038/s41368-023-00228-1

**Published:** 2023-05-30

**Authors:** Yue Zhou, Aki Nishiura, Hidetoshi Morikuni, Wenqi Deng, Toru Tsujibayashi, Yoshihiro Momota, Yuki Azetsu, Masamichi Takami, Yoshitomo Honda, Naoyuki Matsumoto

**Affiliations:** 1 Department of Orthodontics, Osaka Dental University, 8-1 Kuzuhahanazonocho, Hirakata, Osaka, Japan; 2 Department of Physics, Osaka Dental University, 8-1 Kuzuhahanazonocho, Hirakata, Osaka, Japan; 3 Department of Anesthesiology, Osaka Dental University, 8-1 Kuzuhahanazonocho, Hirakata, Osaka, Japan; 4 Department of Pharmacology, Showa University School of Dentistry, 1-5-8 Hatanodai, Shinagawaku, Tokyo, Japan; 5 Department of Oral Anatomy, Osaka Dental University, 8-1 Kuzuhahanazonocho, Hirakata, Osaka, Japan

**Keywords:** Oral diseases, Experimental models of disease

## Abstract

In dentistry, orthodontic root resorption is a long-lasting issue with no effective treatment strategy, and its mechanisms, especially those related to senescent cells, remain largely unknown. Here, we used an orthodontic intrusion tooth movement model with an L-loop in rats to demonstrate that mechanical stress-induced senescent cells aggravate apical root resorption, which was prevented by administering senolytics (a dasatinib and quercetin cocktail). Our results indicated that cementoblasts and periodontal ligament cells underwent cellular senescence (p21^+^ or p16^+^) and strongly expressed receptor activator of nuclear factor-kappa B (RANKL) from day three, subsequently inducing tartrate-resistant acid phosphatase (TRAP)-positive odontoclasts and provoking apical root resorption. More p21^+^ senescent cells expressed RANKL than p16^+^ senescent cells. We observed only minor changes in the number of RANKL^+^ non-senescent cells, whereas RANKL^+^ senescent cells markedly increased from day seven. Intriguingly, we also found cathepsin K^+^p21^+^p16^+^ cells in the root resorption fossa, suggesting senescent odontoclasts. Oral administration of dasatinib and quercetin markedly reduced these senescent cells and TRAP^+^ cells, eventually alleviating root resorption. Altogether, these results unveil those aberrant stimuli in orthodontic intrusive tooth movement induced RANKL^+^ early senescent cells, which have a pivotal role in odontoclastogenesis and subsequent root resorption. These findings offer a new therapeutic target to prevent root resorption during orthodontic tooth movement.

## Introduction

Apical root resorption during orthodontic treatment commonly plagues orthodontists. For example, in some previous studies, the incidence rates of root resorption were reached at 90% and 100%.^1^ Moreover, moderate to severe apical root resorption occurs in 12% to 17% of orthodontic patients, occasionally provoking the unintended clinical side effect, tooth loss.^2^ Nevertheless, currently, there are no effective preventative therapies. Thus, exploring the veiled therapeutic targets based on novel mechanisms is required.

Root resorption is a complex mechanism underlying an orchestra of non-physiological cellular activation and mobilization of numerous cells.^3^ Similar to osteoclasts associated with bone resorption, odontoclasts emerge to resorb root surfaces.^4,5^ The receptor activator of the nuclear factor-kappa B ligand (RANKL)/RANK-related pathway is one of the classic pathways that vigorously promotes the induction and activation of odontoclasts and osteoclastogenesis.^3^ Various cell types, such as cementoblasts and periodontal ligament (PDL) cells, express this ligand.^6,7^ In addition, multiple stressors facilitate RANKL expression, including inflammation, reactive oxygen species (ROS), and non-physiological mechanical stress.^8,9^ For instance, a human immortalized cementoblast cell line and PDL cells significantly express RANKL under stress;^10–14^ Orthodontic tooth movement (OTM) activates cementoblasts to induce RANKL expression.^15,16^ Thus, considering the similarities between osteoclastogenesis and odontoclastogenesis,^17^ osteoporosis drugs (e.g., bisphosphate, which causes direct cell death, and denosumab, an anti-RANKL antibody that prevents osteoclast formation) have been exclusively investigated to treat root resorption.^18,19^ However, thus far, these drugs have not yet reached clinical application.

Cellular senescence is unavoidable for aged cells, resulting in irreversible proliferation arrest, strong mitogenic signals, shortened telomeres, DNA damage, and increased ROS in vitro and in vivo.^20,21^ In particular, DNA damage plays a crucial role in stress-induced premature senescence, evoked by various stressors, such as mechanical, oxidative, radiation, and genotoxic agents.^22–25^ Even in dentistry, ethanol-induced cellular senescence has been reported in cementoblasts and periodontal ligament cells.^26^ Other researchers have also demonstrated that cementoblasts undergo cellular senescence and calcification inhibition in response to mechanical stress stimuli.^27^ Senescent cells generally activate transcription factor cascades, such as p53/p21CIP1, a pathway involved in cell cycle repression, and p16INK4A/RB.^28,29^ Therefore, p21 and p16 are well-used markers for detecting senescent cells.^30,31^ Furthermore, rapid advances in cellular senescence studies have revealed that senescent cells are implicated in various age-related diseases and influencing lifespan by secreting senescence-associated secretory phenotypes, including inflammatory substances^32^ that impair the surrounding tissues.^33^

In 2015, a cocktail of drugs composed of dasatinib (tyrosine kinase inhibitor; used to treat chronic myeloid leukemia) and quercetin (a plant-derived flavonoid) was identified as a specific cell death-inducing agent (i.e., a senolytic) for senescence cells (hereafter dasatinib and quercetin [D + Q]).^34^ Since then, its effectiveness has been confirmed in various diseases, such as diabetes, Alzheimer’s disease,^35^ and osteoporosis,^36^ and for restoring bone regeneration.^37^ Clinical feasibility trials and the development of other senolytics are continuously progressing.^38,39^ Consequently, senescent cells are now recognized as therapeutic targets for various diseases.^20^ Thus, senescent cells are a potential therapeutic target for preventing root resorption. Nevertheless, little is known about the association and localization of senescent cells under OTM and their function in root resorption.

Here, we show that deleterious mechanical stress by OTM induces RANKL-positive (RANKL^+^) senescent cementoblasts and PDL cells, exacerbating root resorption in rat molars using an orthodontic intrusive tooth movement model with an L-loop. Furthermore, we verified that orally administering D + Q remarkably reduced the number of RANKL^+^ senescent cells, attenuating root resorption, indicating that mechanical stress-induced senescent cells are a potential target for root resorption therapy.

## Results

### Apical root resorption model under mechanical stress using an L-loop

**Fig. 1:**
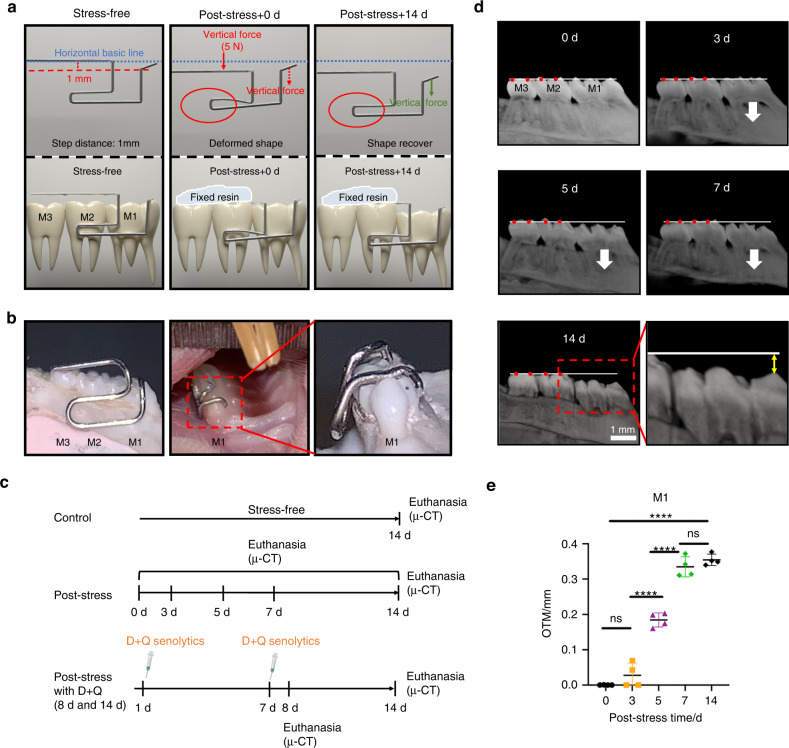
The orthodontic tooth intrusion model in rats. **a** A schematic diagram of the L-loop force system on the orthodontic tooth movement (OTM) model. Horizontal blue lines: L-loop legs at the inactivated stage. After adding vertical force (5 N: red arrow) to the L-loop legs using resin, the left M1 is subjected to the vertical force (green arrow) via leverage effects (red circles). M1: first molar, M2: second molar, M3: third molar. **b** Macroscopic images of the model with the L-loop from the buccal (left) and frontal side (low and high magnification: middle and right images, respectively). **c** An animal experiment flow chart (*n* = 4 per group). D dasatinib, Q quercetin. **d** Reconstructed micro-computed tomography (μ-CT) images of the left maxillary molars. White lines: the baseline for measuring the OTM distance of M1. Yellow two-way arrow: the distance from the cusp of M1 to the basic line. **e** A quantitative analysis of OTM distance from (**d**), represented as means ± standard deviations. ****P < 0.000 1; ns not significant

To mimic apical root resorption, we first established a rat OTM model with a vertically downward L-loop shown in Figs. 1a, b. An L-loop is a conventional apparatus used in orthodontic treatment that generates the desired forces and moments to predictably move the teeth. Consequently, the force directly propagates to the apex and PDL tissue as mechanical stress. The maxillary left first molars (M1s) were subjected to mechanical force (5 N) applied vertically downward continuously for 14 days (Fig. 1c, d). Rats without L-loop treatment were used as a control group (Fig. 1c, Fig. S1). Micro-computed tomography (µ-CT) analysis showed no tooth movement in the control group after 14 days (Fig. S1a), while the M1s significantly moved vertically downward after day 5 with the L-loop, indicating OTM successfully caused mechanical force and stress to the periapical tissues (Fig. 1d, e).

**Fig. 2:**
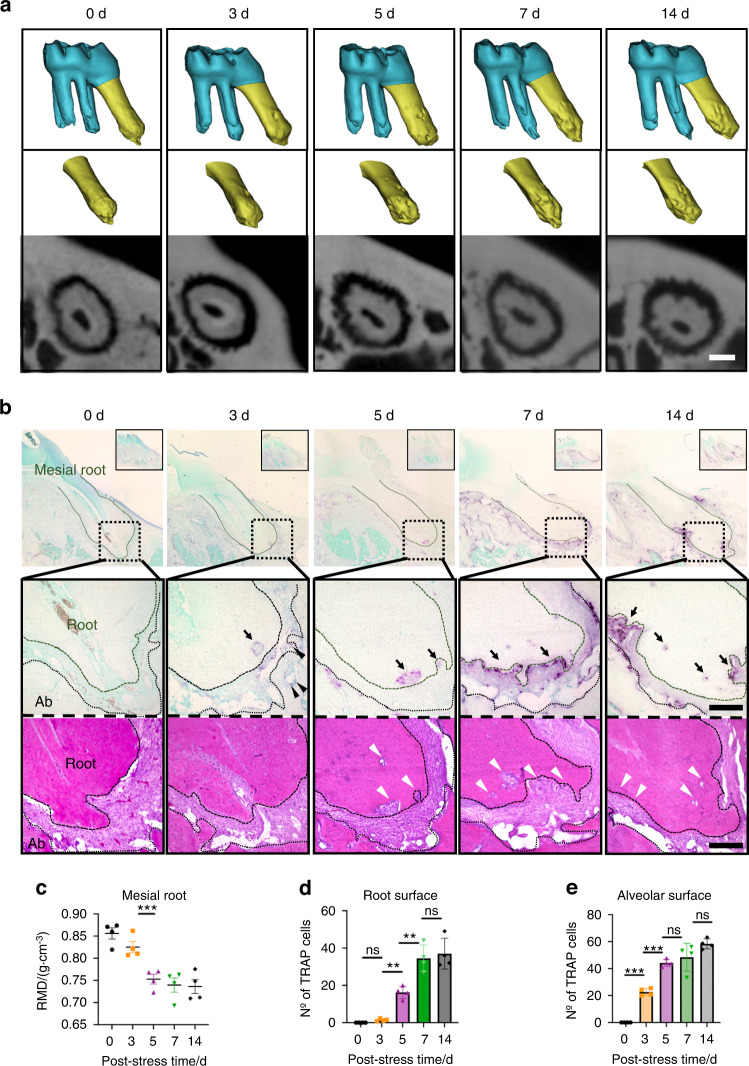
Apical root resorption in the teeth of rats subjected to mechanical stress. **a** μ-CT and three-dimensional reconstruction images of the mesial root apex of the left first maxillary molar. **b** Mesial root images of tartrate-resistant acid phosphatase (TRAP) and hematoxylin-eosin staining after applying mechanical stress. Black arrows: TRAP^+^ odontoclast cells. Black arrowheads: TRAP^+^ osteoclasts. Ab alveolar bone. White arrowheads: root resorption fossa. **c** Root mineral density (RMD) of the mesial root apex. **d** TRAP^+^ odontoclast numbers on the root surfaces. **e** TRAP^+^ osteoclast numbers on the alveolar bone surface. Data are presented as means ± standard deviations. **P < 0.01, ***P < 0.001, ns not significant. Scale bars: 100 μm for (**a**) and 250 μm for (**b**). N^o^ number

Subsequently, to investigate whether the OTM model could cause root resorption, we evaluated the apex of the M1’s mesial root using µ-CT and histological staining, including tartrate-resistant acid phosphatase (TRAP) staining for odontoclast detection. High-resolution μ-CT and three-dimensional (3D) reconstruction images revealed that the morphology of the apical root surface time-dependently changed, becoming irregular under stress (Fig. 2a). Furthermore, the µ-CT quantitative analysis showed that the mean apical root mineral density (RMD) significantly decreased from stress day 5 (Fig. 2c). TRAP staining did not identify odontoclasts (i.e., TRAP^+^ cells) in the control group (Fig. S1b). However, in the OTM group, odontoclasts appeared on the root surface from stress day 3, albeit the numbers were a few (Fig. 2b, black arrows). In contrast, more osteoclasts (TRAP^+^ cells: Fig. 2b, e, black arrowheads) appeared on the alveolar bone surface from stress day 3. Odontoclasts rapidly increased from stress day 5, coinciding with the initiation of root resorption (Fig. 2b, d). Hematoxylin-eosin (H-E) staining images indicated that severe root resorption occurred at the apex from stress days 7 to 14 (Fig. 2b, white arrowheads).

### RANKL^+^ and senescent cells in the periapical tissues under the mechanical stress

**Fig. 3:**
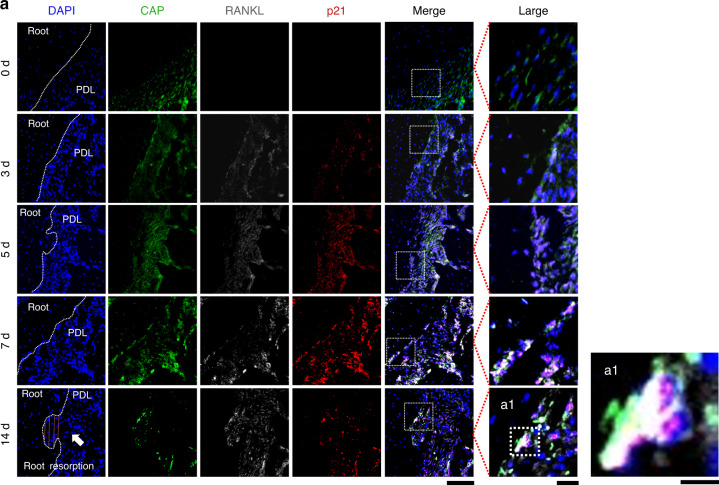
RANKL^+^ cells and p21^+^ senescent cells in periapical tissues under mechanical stress. **a** Immunofluorescence staining and co-localization of cementum attachment protein (CAP) and receptor activator of nuclear factor-kappa-Β ligand (RANKL) with p21 under the 4’,6-diamidino-2-phenylindole (DAPI) counterstaining in periapical tissues after applying mechanical stress. Nucleus: blue (DAPI); CAP: green; RANKL: white; p21: red. Scale bars: 100 and 25 μm for low and high magnification, respectively. White arrows and the orange dashed line in (**a**) indicate the root resorption fossa area. PDL periodontal ligament. **a1** Fluorescence reactive cells in root resorption fossa. Scale bar: 10 μm

**Fig. 4:**
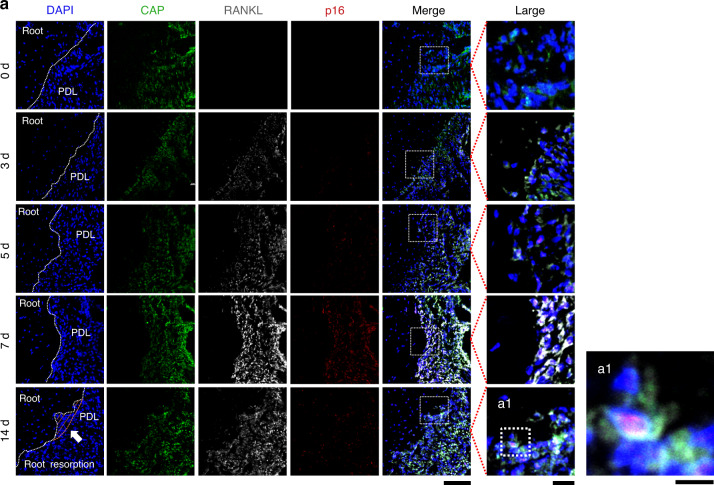
RANKL^+^ cells and p16^+^ senescent cells in periapical tissues under mechanical stress. **a** Immunofluorescence staining and co-localization of cementum attachment protein (CAP) and RANKL with p16 under the DAPI counterstaining in periapical tissues after applying mechanical stress. Nucleus: blue (DAPI); CAP: green; RANKL: white; p16: red. Scale bars: 100 and 25 μm for low and high magnification, respectively. White arrows and the orange dashed line in (**a**) indicate the root resorption fossa area. PDL periodontal ligament. **a1** Fluorescence reactive cells in root resorption fossa. Scale bar: 10 μm

Mechanical stress stimulation induces cellular senescence for cementoblasts and PDL cells^26,27^ and upregulates RANKL expression,^40–42^ which are potentially associated with root resorption. Thus, we verified the localization of cementum attachment protein (CAP)-positive cells expressing senescence markers (p21 and p16) and RANKL using immunofluorescence staining (Figs. 3a, 4a, and S2a). In addition, using Ki-67 (a well-known proliferation marker) and TUNEL staining (usable for DNA damage and apoptosis detection), we confirmed the cellular senescence and DNA damages (Figs. S3 and S4). Cementoblasts and PDL cells secrete CAP; hence, CAP was used to identify them.^43–46^ Hereafter, we define CAP^+^ cells on the root surface as cementoblasts and CAP^+^ cells in PDL as PDL cells.

**Fig. 5:**
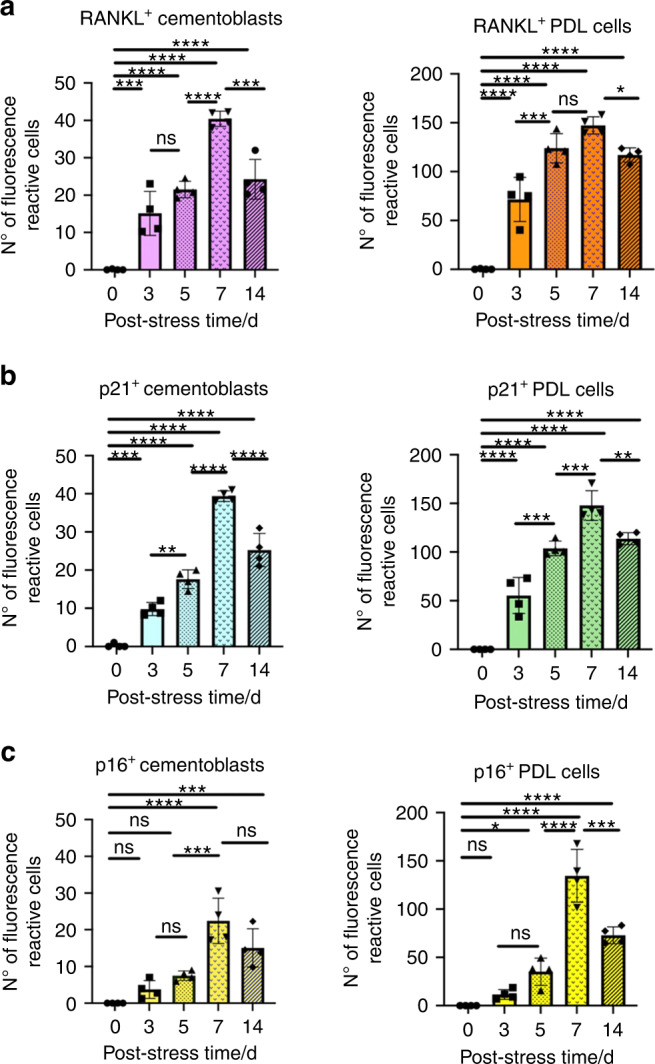
Analyzed the proportion of senescent (p21^+^ or p16^+^)- or RANKL^+^-cementoblasts and PDLs in periapical tissues. **a** The number of RANKL^+^ cementoblasts or RANKL^+^ PDL cells in the periapical tissues. Cementoblasts: CAP^+^ cells on the root surface; PDL cells: CAP^+^ cells in PDL. **b**, **c** The number of p21^+^ or p16^+^ cementoblasts or PDL cells in the periapical tissues. Data are presented as means ± standard deviations. *P < 0.05, **P < 0.01, ***P < 0.001, ****P < 0.000 1, ns not significant. N^o^ number

Figures 3 to 5, Figures S1c and S2a show the spatiotemporal distributions of cells expressing CAP, RANKL, p21, or p16 in the periapical tissues and the quantified numbers. In control group, cementoblasts and PDL cells expressing RANKL or p21 were not observed after 14 days without OTM (Fig. S1c). In contrast, RANKL^+^, p21^+^, and p16^+^ cells appeared in the periapical tissues under mechanical stress (Figs. 3, 4, and Fig. S2a). The number of proliferative cells decreased in parallel to the increase of p21 markers (Fig. S3), whereas the DNA-damaged cells (TUNEL^+^ cells) increased (Fig. S4) under the mechanical stress. RANKL^+^ cementoblasts and RANKL^+^ PDL cells (i.e., RANKL^+^CAP^+^) appeared from stress day 3 and markedly increased on stress days 5 and 7 (Fig. 5a). Similarly, senescent cementoblasts and PDL cells (p21^+^CAP^+^ or p16^+^CAP^+^ cells) increased from stress days 3 to 7 (Fig. 5b, c). Although we could not directly compare the absolute numbers of p21^+^ and p16^+^ cells due to differing sections, we can conclude that the number of p21^+^ cells increased sooner than p16^+^ cells given the statistical difference per section. Among these senescent cells, we found RANKL^+^ cementoblasts and PDL cells in the periapical tissues (Figs. 3 and 4). Figures 3a1 and 4a1 present representative RANKL^+^ senescent cementoblasts in the root resorption fossa.

**Fig. 6:**
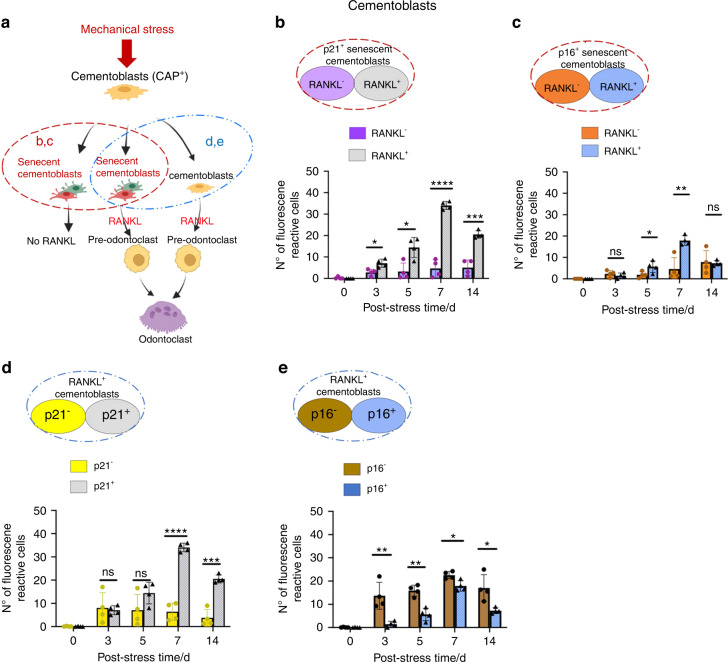
Classification of RANKL^+^ or senescent cementoblasts in periapical tissues under orthodontic tooth movement (i.e., mechanical stress). **a** Schematic diagrams of RANKL^+^ or senescent cementoblasts under mechanical stress. Red broken circles: senescent cells expressing or not expressing RANKL from (**b**) and (**c**). Blue broken circles: RANKL^+^ cells expressing or not expressing senescent markers (p21 or p16) from (**d**) and (**e**). **b**, **c** RANKL expression in senescent cementoblasts (p21^+^/CAP^+^/DAPI or p16^+^/CAP^+^/DAPI). **d**, **e** Senescent marker (p21 or p16) expression in RANKL^+^ cementoblasts (RANKL^+^/CAP^+^/DAPI). Data are presented as means ± standard deviations. *P < 0.05, **P < 0.01, ***P < 0.001, ****P < 0.000 1, ns not significant. N^o^ number

**Fig. 7:**
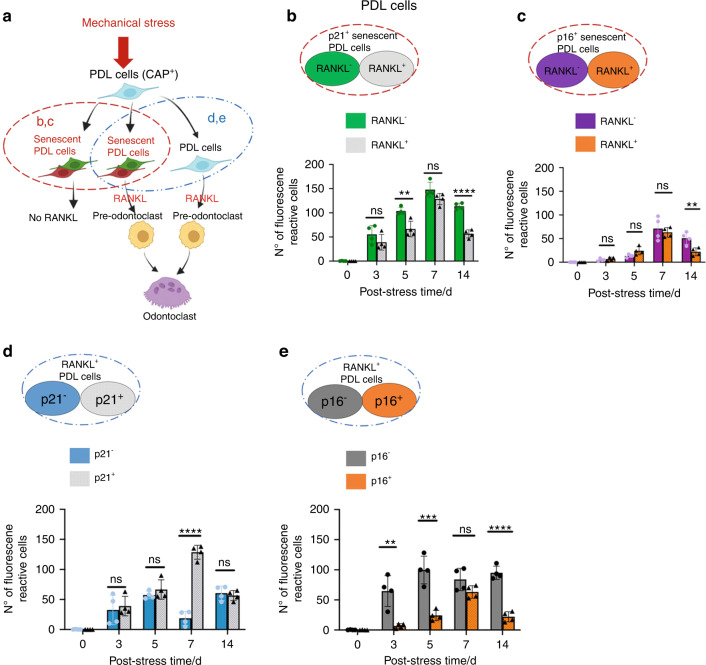
Classification of RANKL^+^ or senescent PDL cells in periapical tissues under orthodontic tooth movement (i.e., mechanical stress). **a** Schematic diagrams of RANKL^+^ or senescent PDL cells under mechanical stress. Red broken circles: senescent cells expressing or not expressing RANKL from (**b**) and (**c**). Blue broken circles: RANKL^+^ cells expressing or not expressing senescent markers (p21 or p16) from (**d**) and (**e**). **b**, **c** RANKL expression in senescent PDL cells (p21^+^/CAP^+^/DAPI or p16^+^/CAP^+^/DAPI). **d**, **e** Senescent marker (p21 or p16) expression in RANKL^+^ PDL cells (RANKL^+^/CAP^+^/DAPI). Data are presented as means ± standard deviations. **P < 0.01, ***P < 0.001, ****P < 0.000 1, ns not significant. N^o^ number

To dissect the character of the cementoblasts or PDL cells (CAP^+^ cells) under mechanical stress, we further classified each cell based on senescent marker expression (p21 and p16) and RANKL (Figs. 6 and 7 for cementoblasts and PDL cells, respectively). In the senescent cementoblasts (p21^+^ or p16^+^; broken red circles in Fig. 6a–c), the RANKL^+^ cells increased over time up to stress day 7, whereas RANKL^–^ cells had only minor changes (Fig. 6b, c); these results suggest that most of the senescent cementoblasts expressed RANKL. Meanwhile, in senescent PDL cells (p21^+^ or p16^+^; broken red circles in Fig. 7a–c), RANKL^+^ and RANKL^–^ senescent cells similarly increased up to stress day 7. Although we could not elucidate the reasons for the above differences, these results demonstrate that senescent cementoblasts and PDL cells sensitively express RANKL in response to mechanical stress in periapical tissue.

Next, we analyzed the proportion of senescent (p21^+^ or p16^+^) cells in RANKL^+^ cementoblasts (broken blue circles in Fig. 6a, d, e). The number of p21^–^ and p21^+^ cells did not differ until stress day 5, then the p21^+^ cells markedly increased from stress day 7 (Fig. 6d). Meanwhile, the number of p16^+^ cells was consistently less than the number of p16^–^ cells up to stress day 14. Of note, p21^–^ cells represent non-senescent cells because p21 is a known early senescence maker.^47^ However, p16^–^ cells are not always non-senescent cells because they also include p21^+^ early senescent cells; p21 and p16 expressions overlap at late senescence.^47^ The propensity of senescent PDL cells under mechanical stress is consistent with the senescent cementoblast outcome (Fig. 7d, e). Collectively, RANKL was primarily expressed in p21^+^ senescent cells rather than non-senescent cells and p16^+^ cells.

### Senescent odontoclasts at the root resorption fossa

**Fig. 8:**
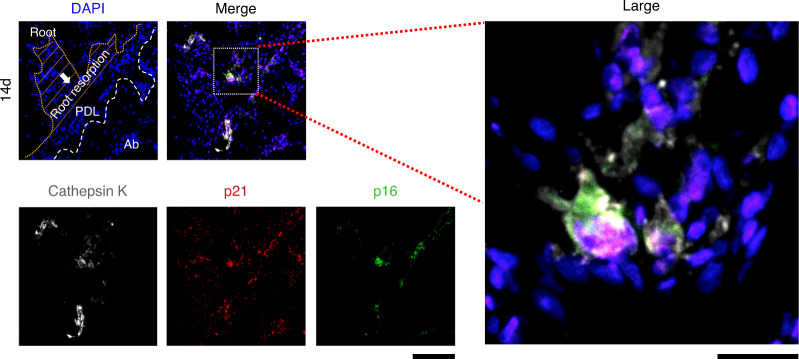
Senescent odontoclasts at the root resorption fossa. Immunofluorescence images of the root resorption sites stained with cathepsin K, p21, p16, and DAPI after 14 days of mechanical stress. Nucleus: blue (DAPI); cathepsin K: white; p21: red; p16: green. Scale bar = 100 μm and 25 μm for low and high magnification, respectively. White arrows and the orange dashed line indicate the root resorption area. Ab alveolar bone

To test whether the odontoclast underwent cellular senescence, we stained the sections of periapical tissues using an antibody for an odontoclast marker (cathepsin K) with senescent markers (p21 and p16). Intriguingly, we found cathepsin K^+^p21^+^p16^+^ multinuclear cells in the root resorption fossa (Figs. 8 and S2b), but not in stress-free groups at day 14 (Fig. S5).

### Oral administration of senolytics prevented root resorption

**Fig. 9:**
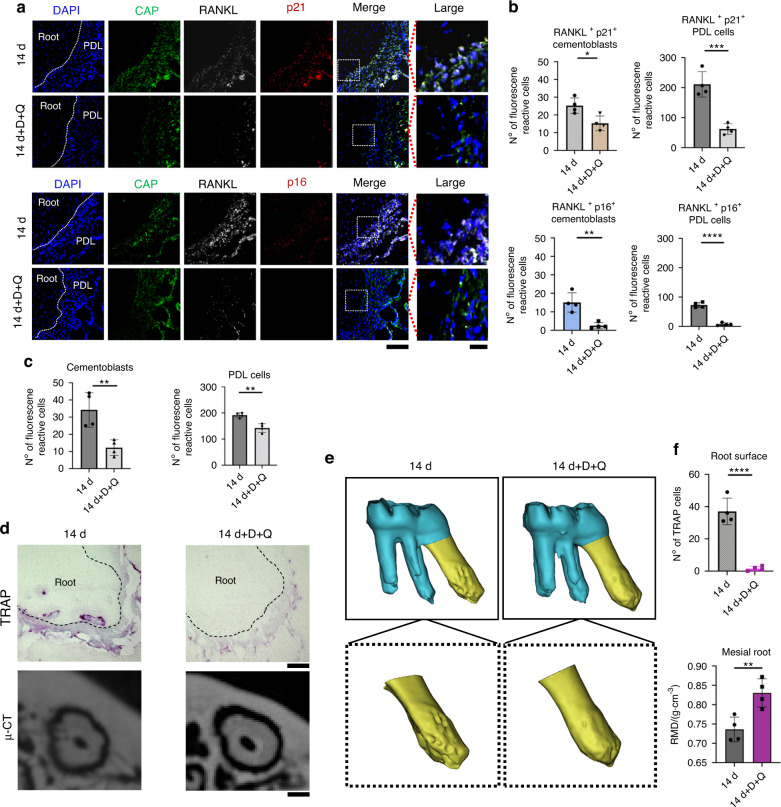
Oral administration of senolytics prevented root resorption. The rats were treated with dasatinib (D) + quercetin (Q) or left untreated at day 1 and 7 during orthodontic tooth movement (i.e., mechanical stress) for 14 days. **a** Immunofluorescence images of the periapical tissues stained with CAP, RANKL, p21, p16, and DAPI. Nucleus: blue (DAPI), RANKL: white; CAP: green; p21 or p16: red. Scale bar = 100 μm and 25 μm for low and high magnification, respectively. **b** The number of RANKL^+^p21^+^ cementoblasts and PDL cells or RANKL^+^p16^+^ cementoblasts and PDL cells in the periapical tissues. **c** Cementoblasts and PDL cells (CAP^+^ cells) in the periapical tissues. **d** TRAP staining and μ-CT images of the mesial root apex. Scale bars: 100 μm for μ-CT images and 250 μm for TRAP staining. **e** Three-dimensional reconstruction images of the mesial root apex of the left first maxillary molar. **f** The numbers of TRAP^+^ cells at the apexes and root mineral density (RMD) of the mesial root apexes. Data are presented as means ± standard deviations. *P < 0.05, **P < 0.01, ****P < 0.000 1. N^o^ number

**Fig. 10:**
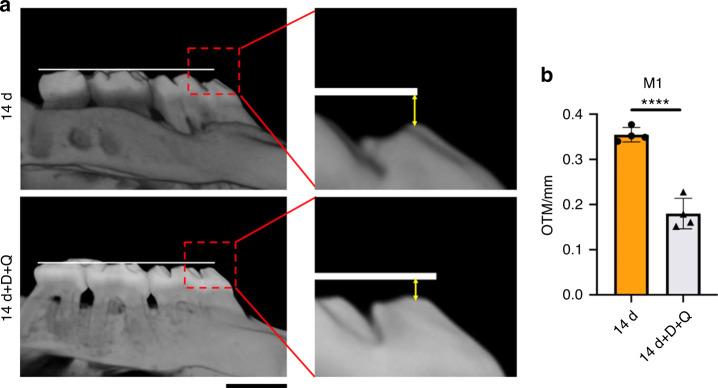
Tooth movement with or without D + Q administration at day 14. The rats were treated with D + Q or untreated at day 1 and 7 during orthodontic tooth movement (i.e., mechanical stress) for 14 days (Fig. 1c). **a** Reconstructed μ-CT images of the left maxillary molars. White lines: the baseline for measuring the OTM distance of M1. Yellow two-way arrow: the distance from the cusp of M1 to the basic line. Scale bar: 1 mm. **b** A quantitative analysis of OTM distance. Data are represented as means ± standard deviations. ****P < 0.000 1

**Fig. 11:**
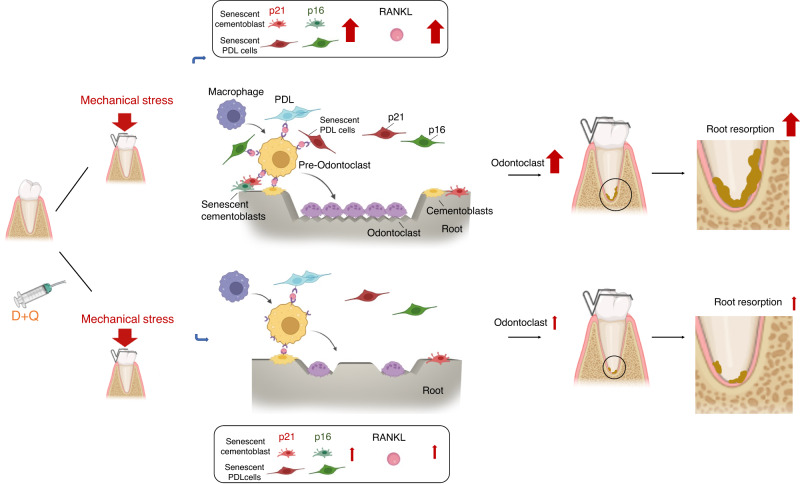
Schematic diagram illustrating root resorption prevention using dasatinib and quercetin after orthodontic tooth movements

To clarify the function of RANKL^+^ senescent cementoblasts and PDL cells for tooth root resorption, we orally administered a senolytic (D + Q) to the rats under mechanical stress on days 1 and 7 (Fig. 1c). The senolytic dose was defined from previous studies.^48^ The number of RANKL^+^ senescent (p21^+^ or p16^+^) cells and cathepsin K^+^p21^+^p16^+^ cells significantly decreased in the D + Q group (Figs. 9a, b and S6) from root surface; the number of Ki67^+^ cells (proliferation cells) was restored, while the TUNEL^+^ cells (apoptotic cells) increased (Figs. S3 and S4). The total number of cementoblasts and PDL cells also decreased in the periapical tissues (Fig. 9c). Furthermore, TRAP^+^ cells and cathepsin K^+^p21^+^p16^+^ multinuclear cells significantly decreased from the root surface (Figs. 9d, f and S6). However, we still identified TRAP^+^ and cathepsin K^+^(but no p21^+^p16^+^) cells on the alveolar bone (Figs. 9d and S6) despite the fact that the senescent cells were decreased (Fig. S6) and were at some parts co-localized near osteoclasts under the mechanical stress (Fig. S7). High-resolution µ-CT images with 3D reconstruction and quantitative analyses determined that the roots had a smoother surface with D + Q administration than without D + Q. The RMD of the proximal apical portion was higher in the D + Q group than in the group without D + Q (Fig. 9e, f). The tooth movement after the D + Q administration decreased to approximately 50% (Fig. 10). The result indicates that orally administering senolytics effectively attenuated the root resorption occurring under mechanical stress by eliminating the RANKL^+^ senescent cells (Fig. 11), whereas further detailed elucidation and cautious improvement are required to advance this therapy for clinical use.

## Discussion

We found RANKL^+^ senescent cells and TRAP^+^ odontoclasts around the apical root with OTM. Repetitive oral administration of D + Q decreased the numbers of those cells and markedly attenuated root resorption. These results indicate that undergoing mechanical stress-induced senescence play a crucial role in root resorption, which is preventable with senolytics.

In this study, we successfully induced vertical forces and mechanical stress to the left maxillary M1s of rats using an L-loop (Fig. 1). OTMs have been mimicked in numerous studies with several experiment models, such as horizontal tooth movement models using elastic chains,^49^ closed-coil springs,^50^ and quad helix-type-appliances,^51^ amongst others.^52^ However, these experimental models mainly have induced horizontal tooth movement. Meanwhile, only a few studies have attempted to develop an intrusion tooth movement model to induce root resorption in the root tips,^52,53^ even though orthodontic treatments often use vertical tooth movement that generates intrusion force. In vertical tooth movement, the apical root and periodontal tissues undergo high mechanical stress, elevating the risk of root resorption^54,55^ After temporary root resorption and subsequent cementum restoration,^56^ the root tip gradually flattens and does not entirely regain its length.^57^ As a result, there is more severity at apical root resorption than lateral root resorption.^58^ Consequently, we prepared an intrusion tooth movement model that proved to be a valuable platform for elucidating the mechanisms underlying apical root resorption. Our prepared model needs only stainless steel wire and is easy to set the loops to the teeth. Meanwhile, the preparation of L-loop shape requires orthodontic skills.

Our µ-CT and histological analyses showed that severe apical root resorption began around stress day 5 (Fig. 2a, b). Interestingly, apical root resorption retarded from bone resorption with OTM (Fig. 2a). In addition, more TRAP^+^ osteoclasts than TRAP^+^ odontoclasts began to appear at the alveolar bone on stress day 3 (Fig. 2b, d, e). Although various researchers have studied odontoclasts and osteoclasts in vivo and in vitro,^59^ the difference in the formation of those two cell types in vivo remains fully unclear. Previous studies have reported that mechanically stimulated cementoblasts express less RANKL than osteoblasts,^10^ and susceptibility to mechanical stimulation differs in cementoblasts and osteocytes.^60^ In addition, the distance from the bone marrow, where monocytes (the origin of odontoclasts and osteoclasts) would be, might be partially associated with the time lag between apical root resorption and bone resorption.

Although p21 and p16 are significant senescence-related markers, their expression kinetics differ.^61^ In our study, p21^+^ cells appeared earlier than p16^+^ cells in the periapical tissues subjected to vertical mechanical stress (Figs. 3, 4, and 5). Previous studies have reported that increments of p21 expression dramatically decreased upon reaching cellular senescence, whereas that of p16 continued for a period and then increased at the end of the cellular life span and the beginning of differentiation.^47^ Thus, some have recently proposed that p21 is an early senescence marker and p16 is a late senescence marker.^47^ In addition, more recent reports indicate that cementoblasts stimulated by mechanical stress express lincRNA-p21,^27^ a critical factor regulating apoptosis and cell proliferation by repressing the translation of target genes through the p53 signaling pathway^62^ (the main pathway regulating p21 expression^61^). Given that p21 was consistently more highly expressed than p16 from day 3 in our study, our data provides evidence that OTM-induced mechanical stress also yields senescent cells in the order of p21 to p16, even in vivo (Fig. 5).

Previous studies have shown that cementoblasts and PDL cells potentially produce RANKL under mechanical stress stimuli in vitro.^12,16^ High RANKL expression promotes the conversion of monocyte-macrophage lineage cells into pre-odontoclasts and increases root resorption activity.^3^ In our study, the number of RANKL^+^ non-senescent (p21^–^) cells and RANKL^+^ senescent (p21^+^) cells did not differ up to stress day 5, but we observed noticeable differences from stress day 7 (Figs. 6d, 7d). These results suggest that RANKL^+^ non-senescent and RANKL^+^ senescent cells contribute to the initiation of odontoclastogenesis. However, the latter possibly plays an essential role in the aggravation or maturation of this formation. Indeed, we eliminated most TRAP and Cathepsin K^+^ odontoclasts by removing senescent cells using senolytics (D + Q) (Figs. 9d, f, and S6).

D + Q administration significantly reduced the numbers of RANKL^+^, p21^+^, and/or p16^+^ cells, leading to root resorption inhibition (Fig. 9a–f). Dasatinib is known to inhibit RANKL expression in bone marrow stromal cells^63^ and osteoclast formation;^64,65^ the drug might directly inhibit RANKL expression from senescent cementoblasts and PDL and odontoclast formation from pre-odontoclasts. Meanwhile, previous studies have shown that mechanical stimulation promotes cells to secrete ROS^66^ and inflammatory cytokines.^67^ Those paracrine factors can cause DNA damage and induce cellular senescence.^68^ In addition, monocyte-macrophage lineage cells exposed to exogenous ROS activate the RANKL signaling cascade, leading to osteoclast formation.^69^ By contrast, quercetin, a D + Q component, has antioxidant and anti-inflammatory effects.^38^ Thus, D + Q treatment may suppress the non-senescent cells to produce inflammatory substances or ROS, which may prevent the induction of senescent cells and the activation of RANKL signaling without causing cell death. However, we administrated D + Q on stress days 1 and 7, and the total number of CAP^+^ cells in the periapical tissues also decreased on stress day 14 (Fig. 9c); TUNEL^+^ cell increased at day 8 (Fig. S4). Therefore, we presume that D + Q had various functions: impairing the induction of cellular senescence in the early phase, causing cell death for the RANKL^+^ senescent cells, and impeding odontoclast formation from pre-odontoclasts, which prevents root resorption in the late phase.

Previous studies have reported that RANKL stimulation causes p21 expression during osteoclast formation.^70^ Thus, we cannot conclude whether odontoclasts undergo cellular senescence only from p21 expression. However, in this study, we found limited number of cathepsin K^+^p21^+^p16^+^ multinuclear cells in the root resorption fossa (Fig. 8), suggesting that the senescent odontoclasts exist under mechanical stress stimulation. Although the functional differences between senescent odontoclast-like cells and other odontoclasts are currently unknown, some cases of apical root resorption have failed to recover for 25 years.^71^ Given the characteristics of senescent cells circumventing apoptosis, the senescent odontoclasts may survive long-term and contribute to the severity of root resorption.

In this study, we demonstrated that the mechanical stress-induced senescent cells induced by OTM contributed to root resorption. However, further detailed examinations are indispensable to explore the mechanisms underlying the relationships among senescent cells, odontoclastogenesis, and root resorption. For example, there is a discrepancy between the number of RANKL^+^ senescent cells and odontoclasts during root resorption (other cells, molecules, etc., may contribute to the odontoclast formation). The long-term association of senescent cells with root resorption remains unclear. Moreover, we must cautiously compare the outcomes in humans and rats. Other conditions, such as distinct administration intervals, durations, and concentrations, would cause different results. Further examination is essential to verify which senolytic agents are applicable for clinical use to evade side effects. Importantly, although our study could not elucidate the detailed molecular mechanisms between RANKL expression and odontoclast formation induced by cellular senescence under mechanical stress using in vitro assays, those data would reinforce the association of senescent cells with odontoclast formation. The mechanisms should be elucidated in detail. However, to our knowledge, this is the first study demonstrating that mechanical stress-induced senescent cementoblasts or PDL cells play a crucial role in the root resorption mechanisms in vivo; the representative senolytic used in this study prevented odontoclastogenesis and root resorption. Furthermore, the spatiotemporal distribution of senescent cells and odontoclasts in periapical tissues under the orthodontic tooth movement was disclosed. These findings may provide new insights into developing new prevention and treatment methods for root resorption, which has been a long-lasting concern for clinicians since orthodontic treatment began.

## Materials and methods

### L-loop preparation

First, a 0.014 inch stainless steel wire (Ormco Corp. Brea, Calif. USA) was bent with light wire pliers, shown in Fig. 1a, b illustrating the initial and activated shapes, respectively. The design is based on the L-loop motif commonly used in clinical orthodontic treatment. The specific design criteria were: (1) L-loops were made with a vertical step of 1 mm between the distal line of the loop and the mesial line of the loop in the initial shape, and (2) L-loops were activated by pushing the distal line down to the same height as the mesial line. The vertical force during activation was 5 N. To confirm the L-loop’s consistency, a vertical test force in the activated state was measured for the L-loop used in the experiment with a universal testing machine device at the Biomaterial research department, Osaka Dental University. The same experimenter repeated the measurement three times to obtain an average test force of 5 N as an OTM model.

### Animal experiments

The local ethics committee at Osaka Dental University approved the animal experiments, and the relevant policies were strictly followed (Approval number: 22-02031). Sprague Dawley rats (male, 15 weeks old) weighing 400–450 g were used for the experimental group to avoid the generation of age-related senescent cells that would affect the experiment. All rats were purchased from SHIMIZU Laboratory Supplies (Kyoto, Japan). The OTM models were fitted as shown in Fig. 1a, b. After general anesthesia using a mixture of three anesthetic agents (butorphanol tartrate: 2.5 mg·kg^-1^, midazolam: 2 mg·kg^-1^, medetomidine hydrochloride: 0.15 mg·kg^-1^), an L-loop was placed on the rats’ left M1 (Fig. 1b). The wires on the occlusal surfaces of the second and third molars were pushed down vertically during attachment, and resin (Kuraray Noritake, Nigata, Japan) was used to fix the wires on the distal portion of the M2 and M3 occlusal surfaces. Then, the L-loop was activated, and a force of 5 N was applied to the M1 occlusal surface. We used 32 rats, divided as follows (4 rats per group; Fig. 1c): 1. No treatment group (Control): euthanized after 14 days; 2. Experimental groups: euthanized on days 0, 3, 5, 7, and 14 after L-loop installation; 3. 8d+D + Q and 14d+D + Q groups: administered D + Q senolytics on days 1 and 7 after L-loop installation and euthanized on day 8 or 14.

### Three-dimensional (3D) tooth root reconstruction by μ-CT

To observe the morphology of OTM and root resorption, rat maxilla samples were scanned by μ-CT (SkyScan 1275, Bruker Co., Billerica, MA, USA) with a voltage of 85 kV, a current of 65 μA, and a high resolution of 1944 × 1546. The 3D data were reconstructed using SkyScan™ CT Analyzer software (version 1.17.7.2) and Mimics (13.1 software Materialise, Leuven, Belgium). Reference lines were drawn along the rats’ left molar’s buccal side at the mesial and distal cusps (Fig. 1d). The vertical distance from the mesial cusp of the M1 to the reference line was deemed OTM (Fig. 1d). The measurements were performed using ImageJ (version: 2.1.0, US National Institutes of Health, Bethesda, MD, USA). The mean RMD of the mesial root apex of the left maxillary M1 was also quantified.^72^

### Histological staining

Samples were fixed in 10% neutral formalin for 24 h. After demineralization in ethylenediaminetetraacetic acid (i.e., EDTA) neutral demineralization solution B (Cat No. LEP2494; Fujifilm Wako Pure Chemicals Corporation) at 4 °C for 2 weeks, the teeth were dehydrated in a sucrose gradient. Frozen sections on all samples were prepared using Kawamoto method.^73^ Serial frozen sections of 10 µm thickness were obtained using cryotome (Leica CM3050S; Leica Biosystems, Richmond, IL, USA). H-E staining of the demineralized sections was performed using standard procedures following a previously reported method.^48^ TRAP staining was performed using the staining kit (Cat. No. 294-67001; Fujifilm Wako Pure Chemicals Corporation) to confirm the odontoclasts and osteoblasts. Images were taken with a BZ-9000 digital microscope (Keyence Corporation, Osaka, Japan).

### Immunofluorescence staining

Decalcified sections were antigen revitalized with 10% Antigen Retrieval Solution HistoVT One (Cat. no. 06380-76, Nacalai Tesque. Inc. Kyoto, Japan), then blocked and permeabilized in 5% goat serum and 0.3% Triton X-100 in phosphate-buffered saline, respectively. Next, sections were conjugated with primary antibodies: anti-p21 polyclonal antibody ALEXA FLUOR® 555 (Cat. No.: bs-10129R-A555, Bioss Antibodies Inc, Woburn, MA, USA, 1:100), anti-p16 polyclonal antibody ALEXA FLUOR® 594 (Cat. No.: bs-23881R-A594, 1:100), anti-p16 polyclonal antibody ALEXA FLUOR® 488 conjugate (Cat. No.: bs-23881R-A488, 1:100), anti-RANKL ALEXA FLUOR® 647 conjugate (Cat. No.: bs-20646R-A647, 1:100), anti-cathepsin K ALEXA FLUOR® 647 conjugate (Cat. No.: SC-48353 AF647, Santa Cruz Biotechnology. Inc, California, USA, 1:100), and anti-cement attachment protein (3G9) ALEXA FLUOR® 488 conjugate (Cat. No.: SC-53947 AF488, 1:100), anti-Ki67 ALEXA FLUOR® 488 conjugate (Cat. No.: 118825, Cell Signaling Technology, Inc., Beverly, Massachusetts, US, 1:100). The sections were incubated overnight at 4 °C and mounted with DAPI-Fluoromount-G®. Then, they were observed under a laser confocal microscope (LSM-700, Zeiss-Microscopy, Jena, Germany).

### TUNEL staining

All of the above TUNEL staining reagents were obtained from the CF™ Dye TUNEL Assay Kits ALEXA FLUOR® 647 (Cat. No. 30074, Fermont, CA, Biotium, Inc.). All sections were stained according to the protocol in kit and mounted with DAPI-Fluoromount-G®. All sections were observed under a laser confocal microscope (LSM-700, Zeiss-Microscopy, Jena, Germany).

### Histomorphometric analyses

The quantitative TRAP, TUNEL staining, and immunofluorescence staining analyses were calculated as follows: the region of interest (ROI) in each section was analyzed under a 10× (TRAP staining) or 20× (TUNEL and immunofluorescence staining) field of view. Then, a layer of cementum cells on the demarcation line of the root edge was selected as the ROI of the root surface per section. The range from the outer layer of cells on the demarcation line to the alveolar bone surface was taken as the ROI of the PDL area. We used ImageJ (version: 2.1.0, U.S. National Institutes of Health, Bethesda, MD, USA) to count the number of TRAP positive cells within the ROI and Fiji2 (version: 1.0, U.S. National Institutes of Health, Bethesda, MD, USA) to count the number of TUNEL positive and immunofluorescence multiplex staining positive cells within the ROI. We tested four samples from four rats (*n* = 4). Each rat was subjected to an independent experiment.

### Senolytics preparation

PEG-200 solution was prepared by dissolving polyethylene Glycol 200 (PEG-200, Cat. No.: ESL3444, FUJIFILM Wako Pure Chemical Co., Osaka, Japan) in MillQ. Dasatinib (Cat. No.: 11498, Cayman Chemical Co., Ann Arbor, MI, USA) and quercetin (Cat. No.: sc-206089A, SCB Santa Cruz Biotechnology Inc., Dallas, TX, USA) were dissolved in the above-prepared PEG-200 solution and mixed thoroughly to make senolytics. Each rat in the post-stress group was orally administered with senolytics at 6.67 mg·kg^-1^ (D) and 66.7 mg·kg^-1^ (Q) on days 1 and 7 after L-loop installation, respectively.

### Statistical analyses

All data were statistically analyzed using Prism 8 (GraphPad Software Co., San Diego, CA, USA). All results were presented as means ± standard deviations. The Student’s t-test was used for comparisons between two groups. A one-way analysis of variance followed by the Tukey multiple-comparison post-test was performed for comparisons between five groups. A *P*-value of <0.05 was considered statistically significant.

## Supplementary information

Supplement file

## Data Availability

All data associated with this study are presented in the paper.
