# Supplementary material for: RANKL+ senescent cells under mechanical stress: a therapeutic target for orthodontic root resorption using senolytics
Source: Int J Oral Sci. 2023 May 30;15:20. doi: 10.1038/s41368-023-00228-1 (PMC10229558; doi:10.1038/s41368-023-00228-1)
Supplement: Supplementary file 1 — Supplement file [file 41368_2023_228_MOESM1_ESM.docx]

**Supporting Information**

RANKL^+^ senescent cells under mechanical stress: a therapeutic target for orthodontic root resorption using senolytics

**Running title:** Senescent cells aggravate apical root resorption

Yue Zhou ^1^, Aki Nishiura ^1^*, Hidetoshi Morikuni ^1^, Wenqi Deng ^1^, Toru Tsujibayashi ^2^, Yoshihiro Momota ^3^, Yuki Azetsu ^4^, Masamichi Takami ^4^, Yoshitomo Honda ^5^*, Naoyuki Matsumoto ^1^

^1^ Department of Orthodontics, Osaka Dental University, 8-1 Kuzuhahanazonocho,
Hirakata 573-1121, Osaka, Japan;

oduzhou@outlook.com (Y.Z.); nishiura@cc.osaka-dent.ac.jp (A.N.); morikuni@cc.osaka-dent.ac.jp (H.M.); dengwenqi95@126.com (W.D.); naoyuki@cc.osaka-dent.ac.jp (N.M.)

TEL: +81-6-6910-1514; FAX: +81-6-6910-1514

^2^ Department of Physics, Osaka Dental University, 8-1 Kuzuhahanazonocho, Hirakata 573-1121, Osaka, Japan;

toru-t@cc.osaka-dent.ac.jp (T.T.)

^3^ Department of Anesthesiology, Osaka Dental University, 8-1 Kuzuhahanazonocho, Hirakata 573-1121, Osaka, Japan;

momota@cc.osaka-dent.ac.jp (Y.M.)

^4^Department of Pharmacology, Showa University School of Dentistry;1-5-8 Hatanodai, Shinagawaku, 142-8555, Tokyo, Japan;

azetsu@dent.showa-u.ac.jp (Y.A.); takami@dent.showa-u.ac.jp (M.T.)

^5^ Department of Oral Anatomy, Osaka Dental University, 8-1 Kuzuhahanazonocho, Hirakata 573-1121, Osaka, Japan;

honda-y@cc.osaka-dent.ac.jp (Y.H.)

Tel: +81-72-864-3013; FAX: +81-72-864-3113

**
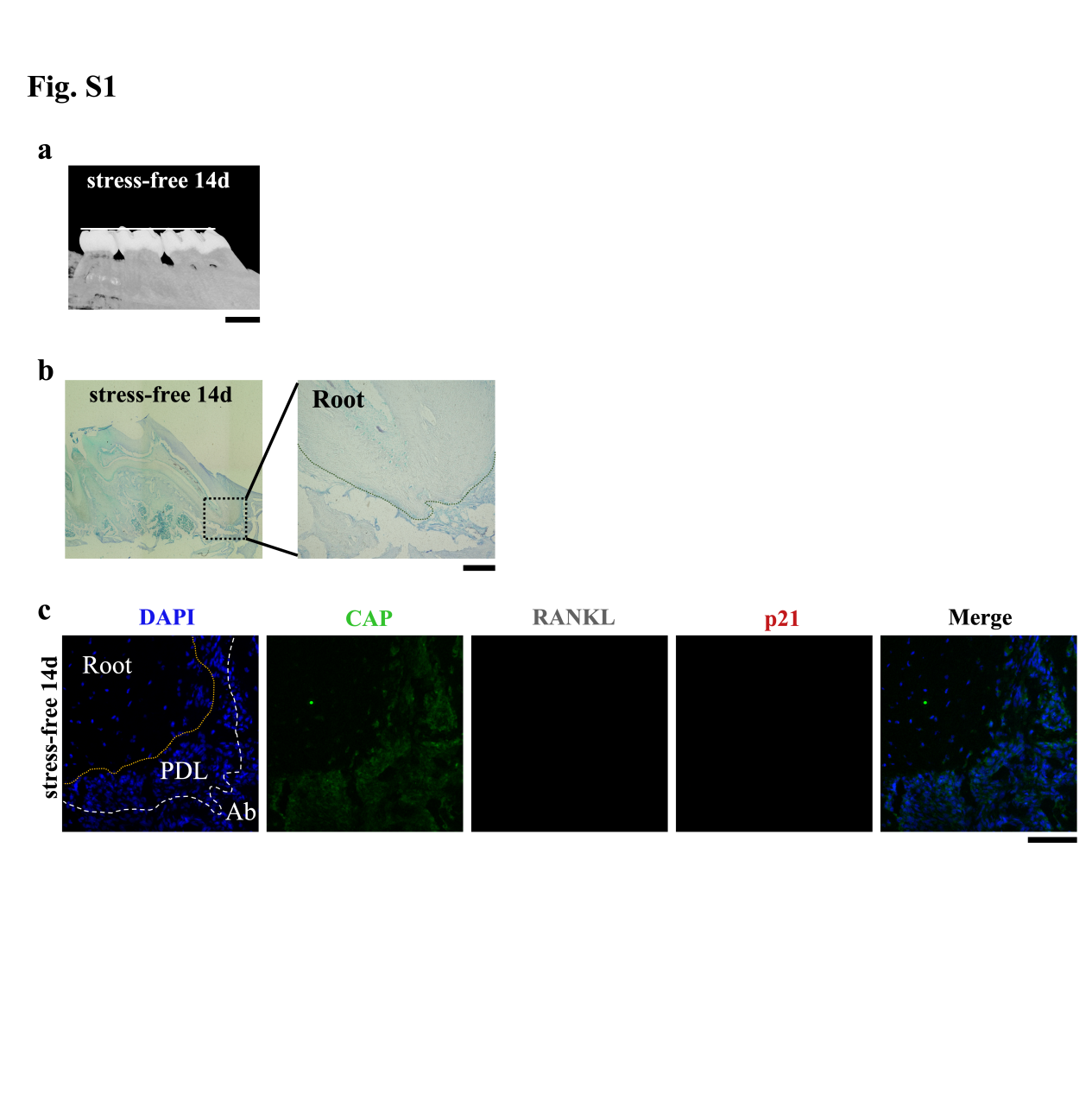
**

**Figure S1** μ-CT datum and histological images of rats without orthodontic tooth movement (OTM) for 14 days. **a** Reconstructed μ-CT image of left first molar at maxilla. White line: the line for confirming the no movement of M1. Scale bar: 1 mm. **b** TRAP staining of periapical tissues without stress. Scale bar: 250 μm. **c** Immunofluorescence images of periapical tissues stained with CAP, RANKL, p21, and DAPI. Nucleus: blue (DAPI), CAP: green, RANKL: white, and p21: red. Scale bar: 100 μm.


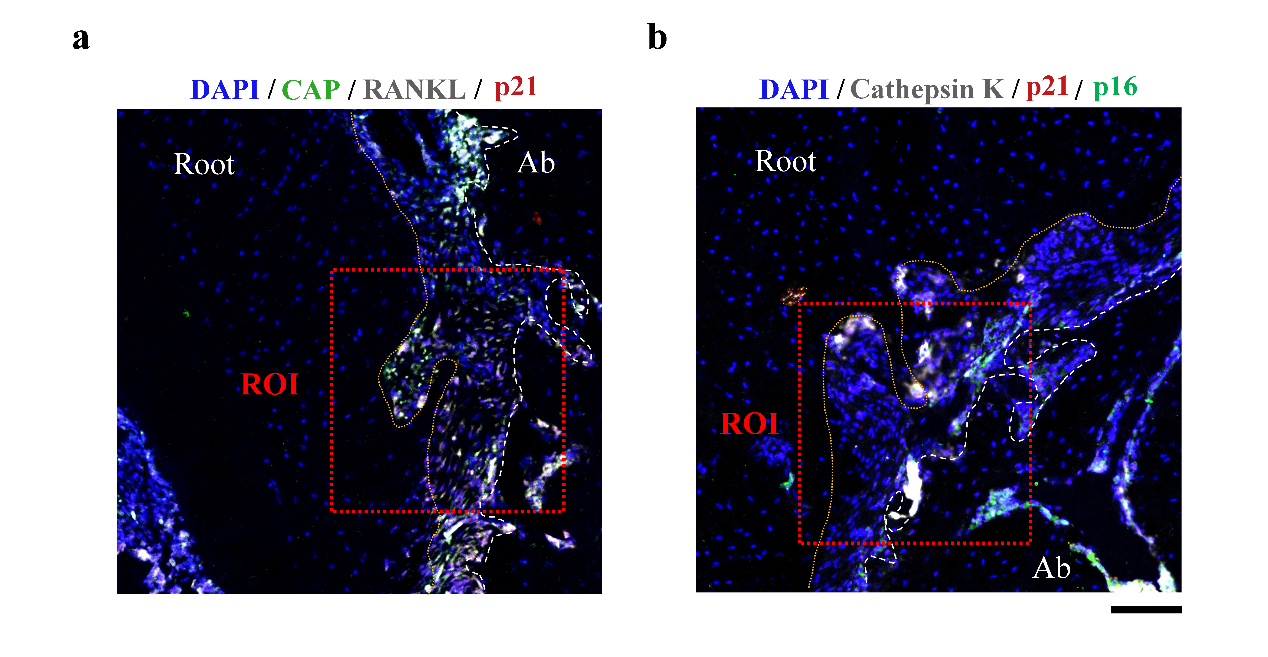


**Figure S2** Representative low-magnification images for Fig.3a1 and Fig.8 to clarify the region of interest (ROI). **a** Immunofluorescence image stained with CAP, RANKL, p21, and DAPI for Fig.3a1. **b** Immunofluorescence image stained with Cathepsin K, p21, p16, and DAPI for Fig.8. Scale bar: 100 μm.


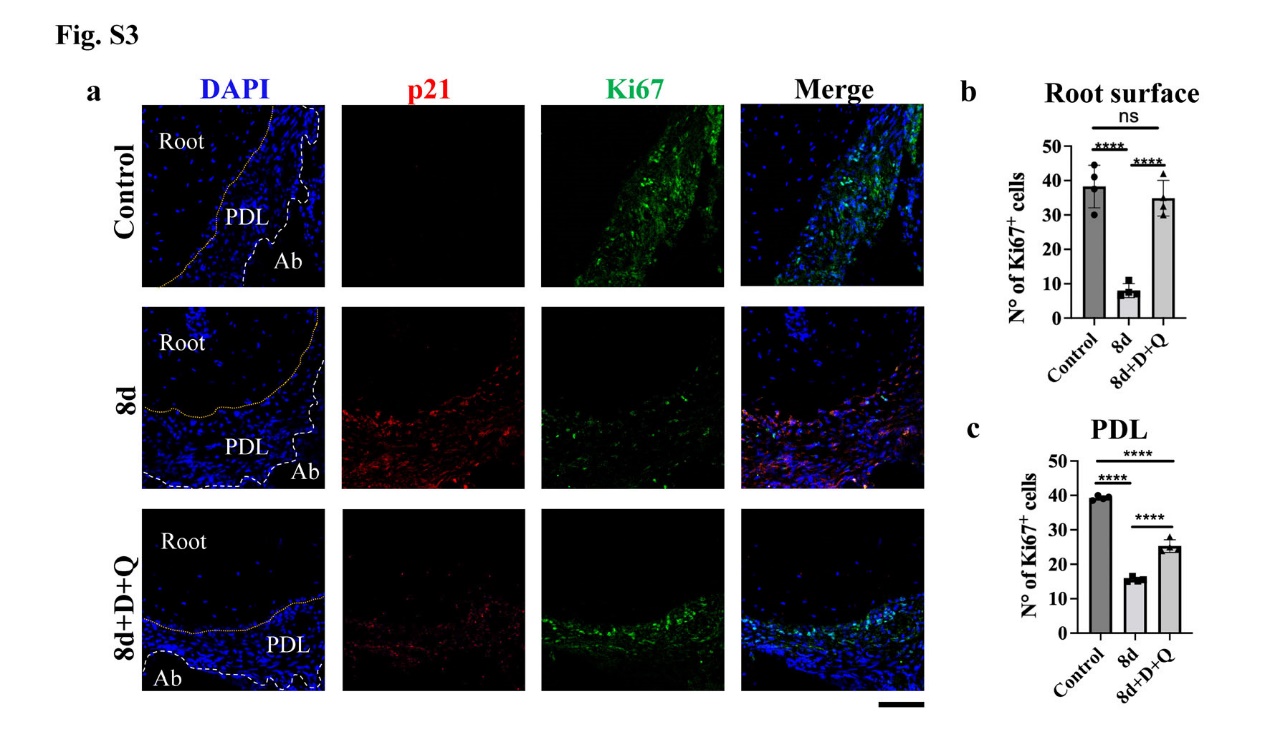


**Figure S3** Proliferating cells in the apical root areas at day 8. Control: no tooth movement and dasatinib (D)+quercetin (Q) treatment. At experimental group, the rats were treated with or without D+Q at day one and seven during orthodontic tooth movement (i.e., mechanical stress) for 8 days. **a** immunofluorescence images of periapical tissues stained with p21, Ki67, and DAPI. Nucleus: blue (DAPI), p21: red; Ki67: green. Scale bar: 100 μm. **b, c** Quantification data of Ki67^+^ cells on root surface or PDL tissues with or without D+Q treatment and orthodontic tooth movement (i.e., mechanical stress). Data are presented as means ± standard deviations. **** p <0.0001, ns: not significant. N^o^: number.


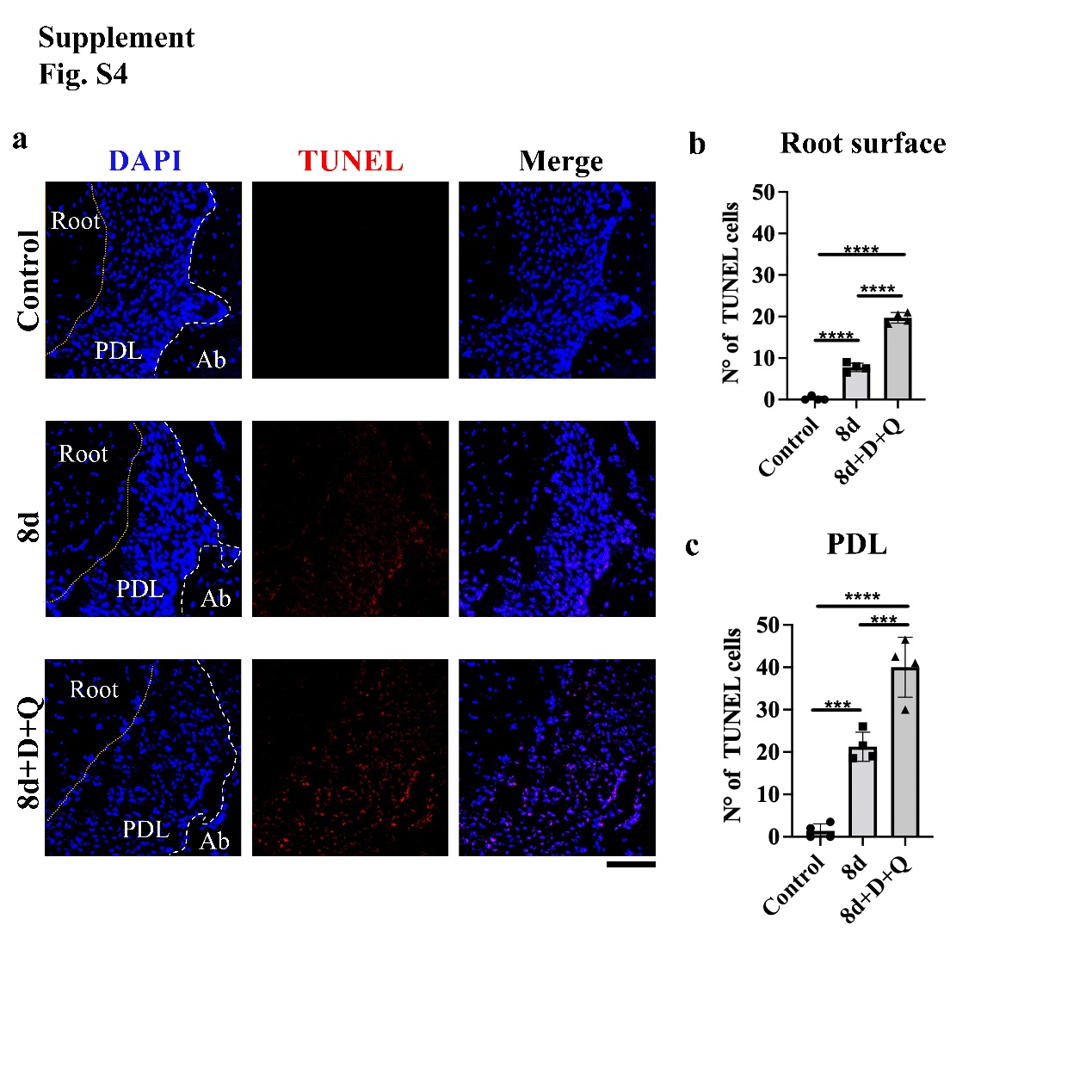


**Figure S4** TUNEL staining for apical root sections to detect DNA damage and apoptotic cells at day 8. Control: no tooth movement and dasatinib (D)+quercetin (Q) treatment. At experimental group, the rats were treated with or without D+Q at day one and seven during orthodontic tooth movement (i.e., mechanical stress) for 8 days. **a** immunofluorescence images of periapical tissues stained with TUNEL and DAPI. Nucleus: blue (DAPI), TUNEL: red. Scale bar: 100 μm. **b, c** Quantification of TUNEL^+^ cells on root surface or PDL tissues under orthodontic tooth movement (i.e., mechanical stress). Data are presented as means ± standard deviations. *** p <0.001, **** p <0.0001. N^o^: number. Ab: alveolar bone.


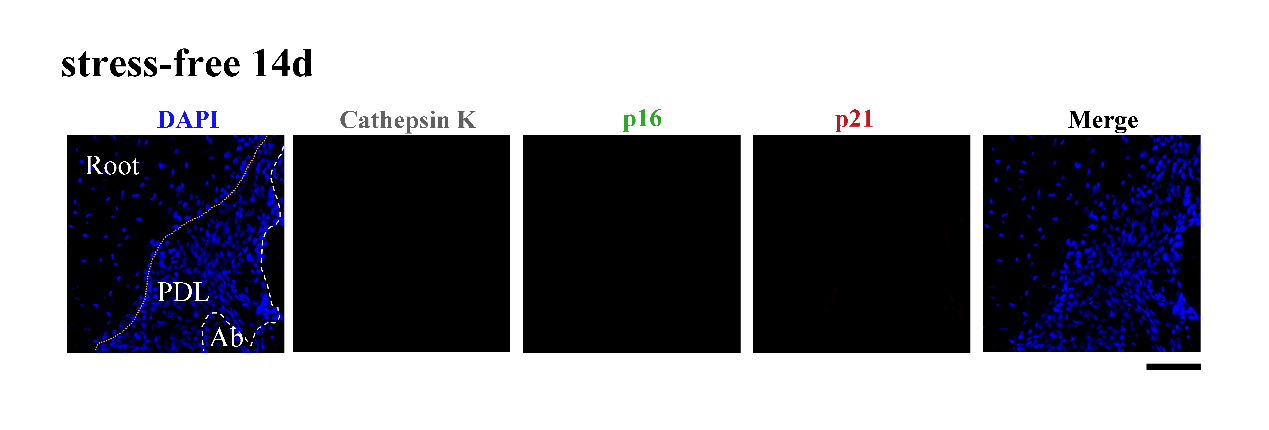


**Figure S5** Immunofluorescence images of periapical tissues stained with Cathepsin K, p16, p21 and DAPI for stress-free 14 days rats. Nucleus: blue (DAPI), Cathepsin K: white, p16: green, and p21: red . Scale bar: 100 μm. Ab: alveolar bone.


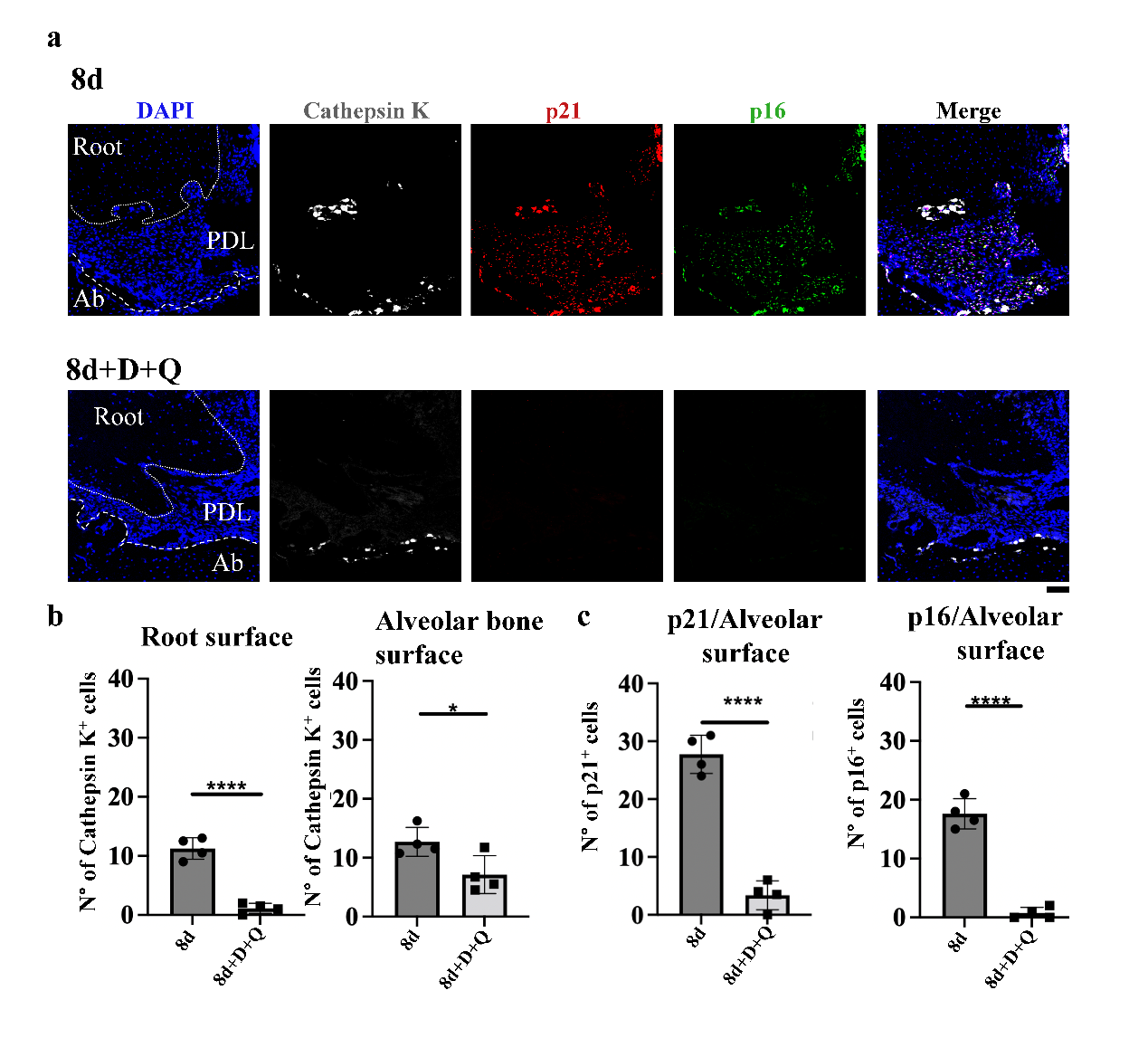


**Figure S6** Senescent cells, odontoclasts, and osteoclasts at periapical tissues at day 8. The rats were treated with or without dasatinib (D) + quercetin (Q) at day one and seven during orthodontic tooth movement (i.e., mechanical stress) for 8 days. **a** immunofluorescence images of apical root tissues stained with Cathepsin K, p21, p16, and DAPI. Nucleus: blue (DAPI), Cathepsin K: white, p21: red, and p16: green. Scale bar: 100 μm. **b** Quantification of Cathepsin K^+^ cells (odontoclast or osteoclast) on root surface or alveolar bone surface. **c** Quantification of p21 and p16 cells on alveolar bone surface. Data are presented as means ± standard deviations. * p <0.05, **** p <0.0001. N^o^: number. Ab: alveolar bone.


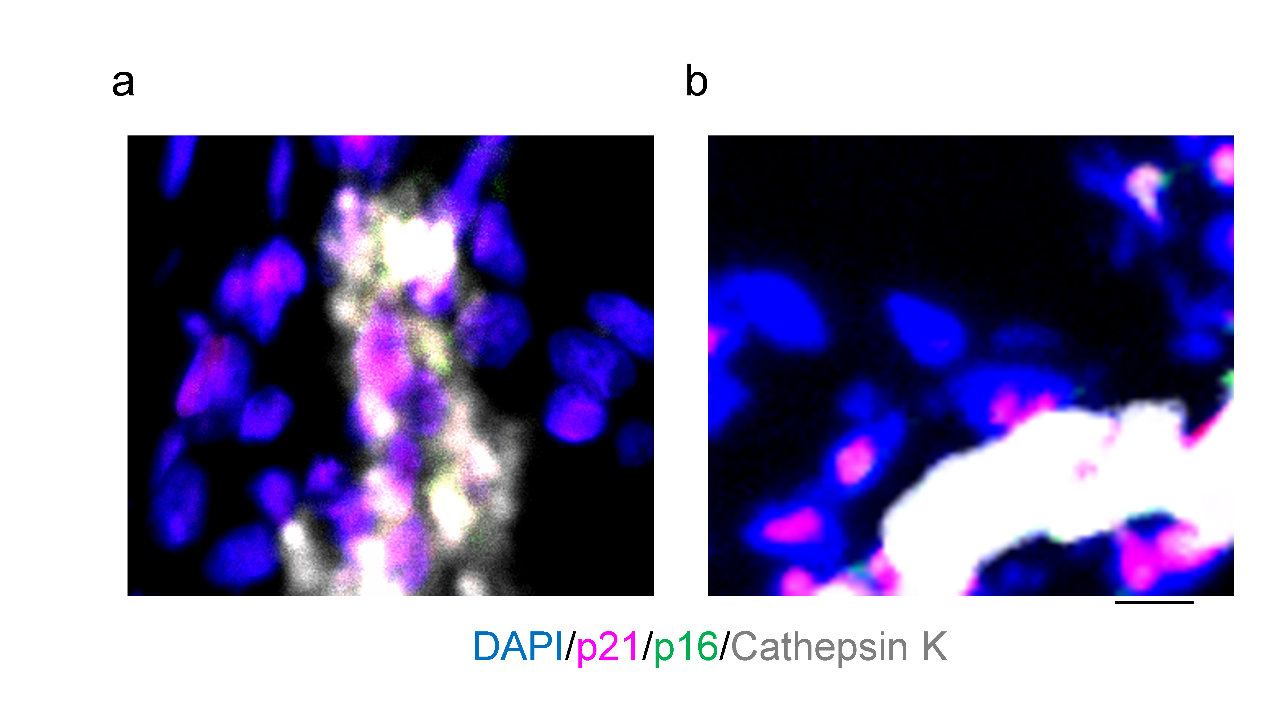


**DAPI/p21/p16/Cathepsin K**

**Figure S7.** Localization of senescent cells near the osteoclasts at alveolar bone surface. **a** high magnification image from Fig.8. **b** high magnification image from Fig.S6. Scale bar: 10 μm.
